# Perceptions of Tuberculosis Patients on Provider-Initiated HIV Testing and Counseling - A Study from South India

**DOI:** 10.1371/journal.pone.0008389

**Published:** 2009-12-21

**Authors:** Beena E. Thomas, Puneet K. Dewan, Sophia Vijay, Aleyamma Thomas, Lakhdir Singh Chauhan, Chandrasekaran Vedachalam, Preetish Vaidyanathan, Soumya Swaminathan

**Affiliations:** 1 Tuberculosis Research Centre, Chennai, India; 2 World Health Organization, Regional Office for South-East Asia, New Delhi, India; 3 National Tuberculosis Institute, Bangalore, India; 4 Central Tuberculosis Division, Directorate General of Health Services, Ministry of Health and Family Welfare, New Delhi, India; McGill University, Canada

## Abstract

**Background:**

The acceptability and feasibility of provider-initiated HIV testing and counseling (PITC) in many settings across Asia with concentrated HIV epidemics is not known. A pilot study of the PITC policy undertaken within the public health care systems in two districts in India offered the opportunity to understand patient's perspectives on the process of referral for HIV testing and linking to HIV treatment and care.

**Methods:**

We conducted a cross-sectional study of randomly selected TB patients registered by the TB control program between July and November 2007 in two districts in south India. Trained interviewers met patients shortly after TB diagnosis and administered a structured questionnaire. Patients were assessed regarding their experience with HIV status assessment, referral for counseling and testing, and for HIV-infected patients the counseling itself and subsequent referral for HIV treatment and care.

**Results:**

Of the 568 interviewed TB patients, 455 (80%) reported being referred for HIV testing after they presented to the health facility for investigations or treatment for TB. Over half the respondents reported having to travel long distances and incurred financial difficulties in reaching the Integrated Counselling and Testing Centre (ICTC) and two-thirds had to make more than two visits. Only 48% reported having been counseled before the test. Of the 110 HIV-infected patients interviewed, (including 43 with previously-known positive HIV status and 67 detected by PITC), 89 (81%) reported being referred for anti-retroviral treatment (ART); 82 patients reached the ART centre but only 44 had been initiated on ART.

**Conclusions:**

This study provides the first evidence from India that routine, provider-initiated voluntary HIV testing of TB patients is acceptable, feasible and can be achieved with very high efficiency under programmatic conditions. While PITC is useful in identifying new HIV-infected patients so that they can be successfully linked to ART, the convenience and proximity of testing centres, quality of HIV counseling, and efficiency of ART services need attention.

## Introduction

The human immunodeficiency virus (HIV) epidemic has the potential to worsen the tuberculosis (TB) epidemic, as has occurred in certain African countries [1]. TB is one of the first opportunistic infections that people living with HIV/AIDS (PLHA) develop. Early HIV diagnosis among TB patients could serve as an entry point for HIV care and treatment, thereby preventing significant morbidity and mortality. This would be expected to improve TB treatment outcomes as well [2], [3].

Few diagnostic tests or screening procedures have engendered as much controversy as the HIV test. With the advent of accurate, rapid HIV test kits and effective treatment, clinicians and public health officials have increasingly called for expanded routine HIV screening [4], [5] and the US Centers for Disease Control and Prevention recently recommended “opt out” HIV screening for all patients aged aged 13 to 64 years in United States healthcare settings [6]. The World Health Organization [7], the Joint United Nations Programme on HIV/AIDS and the International Standards for Tuberculosis Care (2005) recommend providing counseling services and HIV testing for every TB patient in a country with high HIV seroprevalence in the general population [7]–[10]. One approach is routine counselling and testing of patients, also called provider-initiated testing and counseling (PITC) [8], [11].

In India, surveillance for HIV infection amongst TB patients found that HIV seroprevelance ranged widely; among the 15 surveyed districts, between 1% and 14% of TB patients were HIV-infected. Since 2004, the HIV testing approach in healthcare facilities in India has been risk-based referral. With overcrowded clinics and lack of adequate infrastructure to ensure privacy, it is difficult for clinicians to elicit information pertaining to HIV risk behaviours.

A qualitative study from India reported that 69% of TB patients were willing for a HIV test [12]. Willingness, however, does not always translate to actually having a test done. Against this background, prior to large scale implementation across the country, the Indian AIDS and TB Control programmes sought to pilot test PITC and evaluate the feasibility, acceptability and effectiveness of routine referral of TB patients for HIV testing, and of linking HIV-infected TB patients to HIV treatment and care services. The policy was implemented in two districts of south India in July 2007. Alongside the evaluation of referrals, we conducted a cross-sectional survey in a sample of TB patients to explore the acceptance, adherence (to procedures), and associated stigma of routine referral for HIV counseling and testing. This paper describes the findings from this survey of TB patients, focusing on understanding patient's perspectives on the whole process of referral for HIV testing, linkage to HIV treatment and care and the challenges they encountered.

## Methods

### Setting

This was a cross-sectional study done in 2 districts Mysore in Karnataka and Tiruchirapalli in Tamil Nadu, India. The population of both Mysore (2.8 million) and Tiruchy (2.5 million) is spread over 4404 and 6854 sq. km respectively. There are 37 Integrated HIV Counseling and Testing Centres (ICTCs) in Mysore and 27 ICTCs in Tiruchy district. In these 2 districts, physicians and health staff were requested to assess all TB patients for HIV status. If the patient's HIV status was known, that information was to be recorded; if the HIV status were unknown, they were asked to advise the patient to undergo voluntary HIV testing, at the ICTC in the same facility (if available) or at the nearest ICTC. At ICTCs, pre- and post-test counseling is provided for all clients; free HIV testing is conducted by rapid test kits using a 3-test algorithm [13]. Standardized referral forms for ICTC and ART referral were distributed at all health centers. Providers were instructed to use standard referral forms, to encourage patients to return and share the results with the referring provider, and to refer all patients with positive HIV test results to the nearest ART centre.

### Sampling

All TB patients greater than 15 years of age registered by the TB control Program in the two districts were eligible for the study. Participants comprised of two groups. First, TB patients registered from July 2007 to November 2007 in Mysore and Tiruchirapalli randomly selected using systematic sampling of every third registered TB patient over 15 years of age in each district. Second, all TB patients known to be HIV- infected consecutively selected in order to more comprehensively evaluate referrals for ART.

### Data Collection

Data collection was done by trained field investigators with the help of a structured interview schedule. Selected patients were contacted at the treatment site or for those who had a DOTS provider through the treatment supervisors. They were briefed about the study and their written consent was requested, after which the patient was met by the field investigator at a place of their choice. Interviews were conducted after identification of the patient, usually within two months of initiation of anti-TB treatment. The questionnaire covered aspects relating to the patients' socio demographic profile, health seeking behavior, referral for HIV testing, attendance and completion of HIV testing, distance to the ICTC and cost incurred, HIV counseling process, experiences related to stigma and disclosure of HIV results, and their perceptions on being tested for HIV. The distance from the patient's home to the ICTC was recorded as self-reported by patients or—if they were unsure then distance—estimated by the patient with the help of the interviewer based on patient area of residence or bus fare (which increases after 5 km distance). Costs of visiting the ICTC included only patient-reported direct travel costs and travel expenses, and not indirect loss of income.

Patients who were HIV-infected were administered an additional questionnaire covering their referral for antiretroviral treatment, experience at antiretroviral treatment centers as well as the timing of referrals and actual attendance. Data were validated by re-interview of 10% of patients by study investigators, were double-entered in Epi Info 6.04 (Centers for Disease Control, Atlanta, USA and analyzed with SPSS 14.0 (SPSS Institute, Chicago, USA.

### Ethical Approval

This activity was reviewed and approved by the institutional ethics committees of the National Tuberculosis Institute, Bangalore and Tuberculosis Research Centre, Chennai and Indian Council of Medical Research (ICMR).

## Results

**Figure 1:**
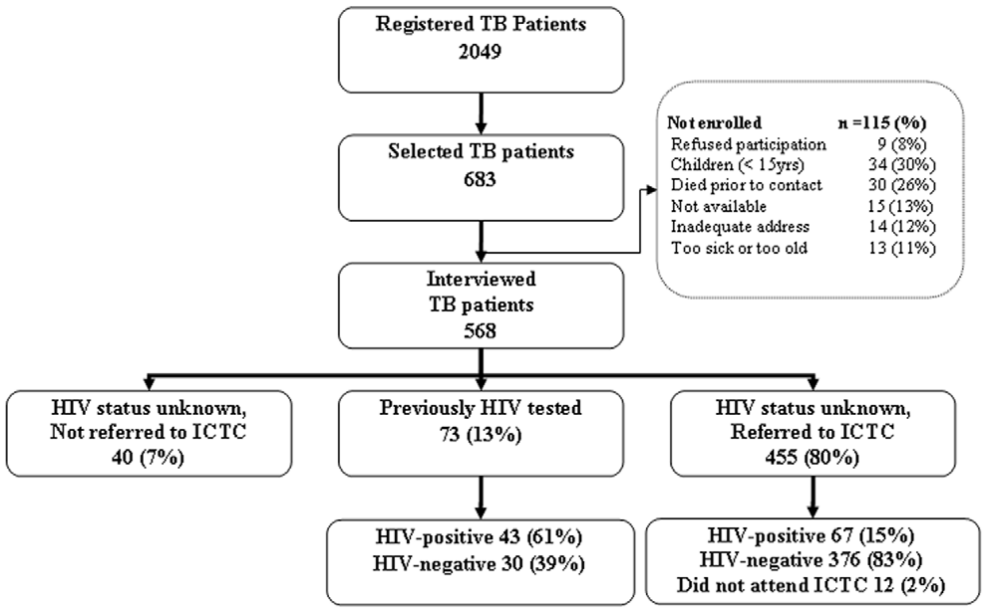
Selection and enrollment of tuberculosis patients and HIV status of interviewed tuberculosis patients at time of tuberculosis diagnosis. Mysore and Trichirapalli, 2007. Abbreviations: ICTC = integrated HIV counseling and testing centers, TB = tuberculosis.

From July 2007 to Nov 2007, 2049 TB patients aged 15 years or older were registered in Mysore and Tiruchirapalli and a total of 683 patients were selected for interview. These included 361 patients from Mysore and 322 patients from Trichy. From the 683 patients selected, 568 (83%) patients completed their interviews. The reasons for non-completion/non-availability for interviews included patient illness, inadequate address records or death (Figure 1). There was no significant difference between the 568 respondents and the 115 non respondents with regard to their median age, sex or residence.

### Patient Characteristics n = 568

Among interviewed patients, 80% of the patients were between the ages of 15 to 54 years; the median age being 38 years. Approximately 50% were from rural areas and two-thirds were literate (Table 1). The overall average monthly income of the employed respondents was approximately Indian Rupees (Rs.) 1500 (∼USD$30).

**Table 1:** Basic characteristics of interviewed tuberculosis patients.

Characteristic	Number (N = 568)	%
District of Residence		
Mysore	317	56
Tiruchirapalli	251	44
Age Distribution		
15–24 years	101	18
25–34	120	21
35–44	127	22
45–54	106	19
55–64	76	13
> = 65	38	7
Sex		
Male	379	67
Female	189	33
Residence		
Rural	317	56
Urban	176	31
Semi-urban	75	13
Marital Status		
Single	109	19
Married	397	70
Widow	53	9
Separated	9	2
Occupation		
Unemployed	272	48
Employed	296	52
Education		
Illiterate	207	36
Literate	361	64
Perceptions of TB patients on need for HIV testing		
Yes	347	61
No	28	5
Don't know	193	34

### Details of Referral and Experiences in Reaching ICTC and at ICTC (n = 443)

Of the 568 interviewed TB patients, 73 (13%) reported that they had already been previously tested for HIV before TB diagnosis, among whom 43 were HIV-infected. Of the 495 patients without known HIV status, 455 (92%) reported being referred for HIV testing by the time of interviews. Of these 455, 67 (15%) were subsequently found to be positive for HIV. Hence there were a total of 110 HIV-infected respondents among the 568 interviewed TB patients.

Among the 455 who were referred, 443 (97%) reported that they attended an ICTC and had HIV testing done. Twenty-three percent of the respondents walked the distance to the ICTC, covering a distance of anywhere between one and 5 kilometers (**Table 2**). More than 50% had to travel more than 6 kilometers, and more than half had to spend more than Rs. 25 (∼USD$0.50) to go to the ICTC.

**Table 2:** Reported experiences during referral for HIV-testing among tuberculosis patients who reported attending an integrated counseling and testing centre (ICTC).

Characteristic	Number N = 443	%
Distance from Home to the ICTC centre (km)		
<1	11	3
1–5	197	46
6–10	84	19
11–20	94	22
> = 21	45	10
Mode of Transport to ICTC		
Walk	100	23
Bus	301	69
Train	1	-
Cycle	12	3
Two-wheeler	24	5
Total Expenses (Indian Rupees) for each ICTC visit		
Nil	25	6
1–10	81	20
11–25	87	22
26–50	102	25
51–100	71	18
> = 101	35	9
Number of visits made to ICTC		
1	261	59
2–4	163	37
5	6	1
Not undergone pre-test counseling before HIV testing	230	52
Informed by counselor to disclose HIV result to M.O	247	56
Disclosed HIV test result to referring physician directly	333	75
Asked to provide written consent	362	82
Asked to take test in front of other patients	104	24
Blood test result collected	406	92
No specific difficulties at ICTC	443	96

One hundred and sixty nine (38%) patients had to make more than two visits to the ICTC to complete testing. Among those patients tested, 230 (52%) of the patients reported that they had not undergone pre-test counseling, which is required as per National AIDS Control Programme guidelines. While local guidelines asked counselors to inform patients that they should self-disclose their HIV results to their treatment provider, 247 (56%) of patients reported being informed by the counselor to do so. Regardless, 333 (75%) of patients reported voluntary disclosure of HIV results. These included patients who were informed to disclose their results as well as those who informed the medical officer directly on their own. While national guidelines specify privacy for counseling and written consent for testing, 104 (24%) said they were asked to have the test in front of other patients and 362 (82%) said that they were asked to provide a written consent. Four hundred and six (92%) of the 443 patients had collected the results of their HIV test.

### Referral to the ART Center among HIV-Positive Patients (n = 110)

**Figure 2:**
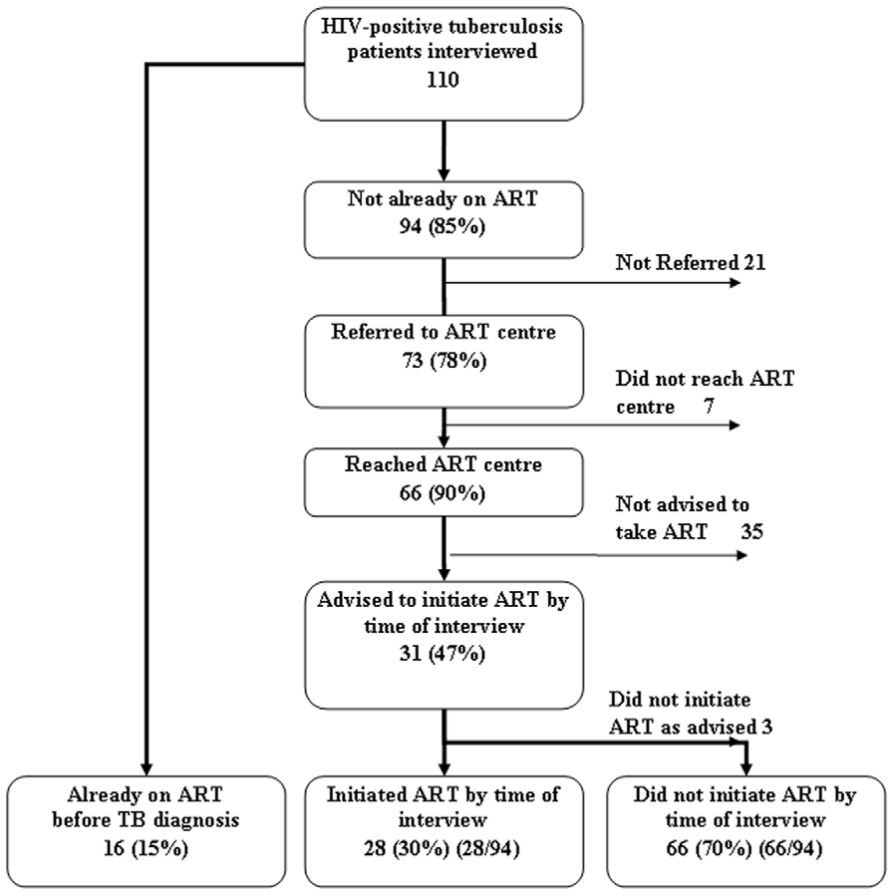
Referral to ART center and initiation of ART among HIV- infected TB patients.

Of the 110 HIV-infected patients, 16 (15%) were already on ART at the time of TB diagnosis. Of the remaining 94 patients not yet on ART, 73 (78%) patients reported being referred to the ART centre, and 66 (90%) reported that they reached the centre (Figure 2). Subsequently, 31 (47%) patients reported that they were advised to start ART and 28 of these were initiated on ART.

Of the 66 who reached the ART centre, 28 (42%) patients went to the ART centre after a gap of more than two days and 47 (71%) patients traveled for more than 10 km to get there. Twenty-three (35%) patients reported that the total expenses incurred to reach the ART centre exceeded one hundred rupees (∼USD$2). Few patients were referred using the provided referral form, and use of verbal-only referrals was common. Some of the constraints expressed by patients regarding their experience in ART centre referral and visitation included distance to reach ART center, long waiting time, overcrowded clinics, too many personal questions and too many visits. The number of visits may have been affected by operational challenges in obtaining CD4 count testing—which at the time of this study and in these settings was done only on specific days—and for collection CD4 results thereafter (data not shown).

Of the 110 HIV-infected patients interviewed, 44 (40%) were on both ATT and ART including both the 16 who were on ART at the time of TB diagnosis, and the 28 patients who were newly-initiated on ART after TB diagnosis. Among these 44, 37 (84%) said they had no problems in taking TB and anti-retroviral treatment (Table 3). Some challenges, however, raised by patients included the pill burden, inability to go to work, inconvenient timings, adverse reactions, and distance to the ART centre.

**Table 3:** Experiences reported by HIV positive TB patients on linkage to HIV care and antiretroviral treatment.

	Number N = 110	%
ART statues at time of TB diagnosis		
Taking ART	16	15
Not Taking ART	94	85
Referral to the ART centre (n = 94)		
Not Referred	21	22
Referred to but did not reach ART centre	7	8
Referred and reached ART centre	66	70
Reported mechanism of referral (n = 66)		
Referral from	36	55
OPD ticket	15	23
No paper referral – verbal only	7	11
Others	8	12
Number of days a taken to reach ART centre (n = 66)		
Same day	11	17
Next day	27	41
Over 2 days	28	42
Distance traveled to ART centre (km) (n = 66)		
1–5	11	17
6–10	8	12
11–20	6	9
> = 20	41	62
Total expenses incurred in travel to ART centre (Indian Rupees)		
0–10	6	9
11–25	11	17
26–50	18	27
51–100	8	12
> = 101	23	35
Problems at ART Centre (n = 66)		
Yes	9	14
No	57	86
ART initiation(n = 66)		
Patient reported not being advised to start ART	35	53
Advised to take ART but did not initiate	3	5
Advised and initiated ART	28	42
Problem in talking ART and ATT both (n = 44)		
Yes	7	16
No	37	84

### Knowledge about HIV Diagnosis amongst HIV-Positive Patients (n = 110)

Participants were directly asked by the field investigators both if they had TB and if they had HIV infection. Of the 110 HIV- infected TB patients, 95 openly acknowledged they had TB, but only 15 (14%) disclosed their HIV status to the interviewer. Fifty-two (47%) of the 110 HIV infected patients reported that they did not know whether or not persons with HIV infection were more vulnerable to TB, while 43% of patients correctly identified HIV as a risk factor for TB.

## Discussion

This study provides the first patient-level evidence from India that routine, provider-initiated voluntary HIV testing of TB patients can be achieved with very high efficiency under programmatic conditions. The majority of TB patients (92%) reported being referred for HIV testing, and 97% of these completed HIV testing with the majority of patients collecting their results, despite the distance to the testing centres and incurring financial difficulties.

It is equally encouraging that nearly two-thirds of the TB patients who were ultimately identified as HIV-infected were newly diagnosed through provider-initiated HIV testing. Our findings from patient interviews strengthen and extend those reported from record review in the same setting, which found that 70% of TB patients had documented evidence of HIV status [14]. These findings from India add to the accumulating body of evidence globally demonstrating that PITC is not only feasible, but that it can be accomplished with very high efficiency in programmatic settings [15], [16].

If routine referral has to be successfully and widely implemented, care needs to be taken to address some of the concerns which have been brought out from this study. Many patients incurred substantial expenses as they had to travel to the ICTC multiple times to complete HIV counseling and testing and to receive their test results. Approximately one-third of the patients did not know if they should be screened for HIV; we speculate that this finding relates to patients' self-perception as low-risk for HIV infection, similar to that reported other studies in vulnerable populations in North America [17]–[19]. The stigma associated with being HIV positive seemed to loom large among the patients, with only 15% of the HIV-infected TB patients openly admitting to the interviewers that they had HIV, despite willingly answering questions about HIV testing and ART referral. Furthermore one-fourth of the patients said they were asked to have the test in front of other patients, and only two-thirds of the patients were informed that they need to disclose their results to the medical officer. It is also worrisome that more than half of the patients reported that they were not counseled before HIV testing; this finding suggests that patient services in these settings were not conducted as per national guidelines, and warrants further evaluation. As reported in other studies poorly motivated personnel, poor communication, and shortage of human and financial resources are barriers to successful joint TB-HIV interventions [20]–[22].

Health providers at all levels including medical officers, laboratory technicians, pharmacists, nurses and counselors need to be sensitized on the need for this integration to ensure that PITC for TB patients is done effectively [23]. Attention to the monitoring and supervision of quality of counseling services may improve compliance with guidelines and improve the overall experience of patients. As the number of patient who are HIV tested increases, patient load may further affect counseling quality. While it is important that the process needs to be strengthened, testing of large numbers of clients may not be possible through the traditional ICTC alone. The ICTC alone (usually located in larger health facilities such as medical colleges, district health centres or block primary health centres) and alternative strategies like point-of-care HIV testing by laboratory technicians or nurses at the primary care clinic needs to be explored, especially in remote areas. Advocacy by the media could also help in raising public awareness and promoting the importance of routine testing for HIV, as reported in a study from Botswana [24].

With regard to HIV treatment services, amongst the interviewed HIV-infected TB patients the majority reported that they were referred to the ART centre and reached there, despite substantial distances and expenses incurred. There was an unexplained gap, however, between those who reported reaching the ART centre and those initiated on ART. By the time of interview, approximately 30% of the patients reported being treated with both ART and ATT. Thirty-seven percent of patients who reached the ART centre reported that they were not advised ART; whereas in the parallel study of medical records more than 70% of HIV-infected TB patients should have been classified as eligible for ART [14]. This discrepancy suggests the need to evaluate systems for ART evaluation and initiation. The finding of relatively low ART uptake is consistent with a study from Thailand which reported that acceptance of provider-initiated HIV testing among tuberculosis patients was being high but the provision of HIV services disappointingly low [15]. Nearly one-fourth of patients did acknowledge specific challenges, including distance, frequency of visits required, and expenses incurred. These findings suggest possible pathways to improve linkages of HIV-infected TB patients to HIV treatment, such as improved counseling, transportation assistance, streamlining ART evaluation processes and decentralizing ART. Monitoring and evaluation services on a regular basis should be ensured as potential means to improve ART uptake, and by extension improve overall TB and HIV outcomes and patient well-being.

### Limitations

Our results were drawn from patients in only 2 districts in South India, and two-thirds of the respondents were males, and are not expected to apply to all settings or all patient populations. In interpreting these findings, one should be aware of the effect the presence of the research team may have had, as pointed out in a study in Malawi, which showed that acceptability for routine HIV testing among TB patients was above 90% under research conditions but lower (59%) under routine care conditions [25]. In this setting, however, interviewers did not interact with patients till well after their TB diagnosis, hence these findings are essentially from routine care. A larger parallel study in the same area based on clinical and programme records found that 72% of patients without known HIV status were referred for HIV testing, which is substantially below what we documented in this patient population (92%) [14]. This may be attributable to weaknesses in documentation of HIV-testing, and we would consider our patient interviews more likely to reflect the underlying reality of HIV testing in TB patients than medical records.

### Conclusion

Provided-initiated voluntary HIV testing of TB patients is acceptable, feasible and can be effectively implemented under routine programmatic conditions. While implementation of this policy was vey effective in identifying new HIV-infected patients and referring them to care, counseling services did not consistently offer services as per national guidelines. Amongst HIV-infected TB patients, ART initiation was lower that expected. Providers referring patients to ART services should ensure completion of ART evaluation. Decentralization of ART services—currently planned by NACP—may also improve ART uptake.
